# Ordered lamellar supermicroporous titania templating by rosin-derived quaternary ammonium salt

**DOI:** 10.1371/journal.pone.0180178

**Published:** 2017-06-30

**Authors:** Fei Song, Peng Wang, Shangxing Chen, Zongde Wang, Guorong Fan

**Affiliations:** College of Forestry, Jiangxi Agricultural University, Nanchang, P.R. China; University of Sheffield, UNITED KINGDOM

## Abstract

By using dehydroabietyltrimethyl ammonium bromine (DTAB), a novel rosin-derived quaternary ammonium salt, as template and peroxotitanium acid as precursor, ordered lamellar supermicroporous titania has been synthesized via a hydrothermal process. The template agent:titanium source molar ratio in the synthesis system and the hydrothermal temperature have great impact on the microstructure characteristics of the samples. The increase of DTAB:TiO_2_ molar ratio from 0.04:1 to 0.10:1 is favorable to the increase of regularity of pore structures, but has no significant effects on the crystalline structures. The increase of the hydrothermal temperature from 343 to 393 K can induce an increase in crystallinity of the samples. However, the exorbitant hydrothermal temperature will reduce the regularity of pore structures. When the mole ratio of DTAB:TiO_2_ is 0.10:1 and the hydrothermal temperature is 373 K, the as-synthesized sample possesses pore structure with the highest level of long-range order, as well as pore wall with semicrystallized anatase phase. The pore size and the pore wall thickness are about 2.0 nm and 1.0 nm, respectively.

## Introduction

Since M41S type molecular sieves were firstly synthesized in 1992,[1, 2] mesoporous materials have received enormous attention. Through the soft-templating route, different kinds of mesoporous materials were successfully fabricated.[3–9] Such materials possessing pore sizes larger than 2.7 nm are of great interest as they can lead to fast diffusion for the large-molecules that cannot access the pores of microporous zeolites. However, the pore sizes of mesoporous materials are too large to exhibit effective shape-selectivity in most of separation or catalytic processes.[10] Supermicroporous materials with pore sizes ranging from 1.2 to 2.7 nm may be more suitable for such tasks.[11] Because the hydrophobic chain lengths of surfactants have great effects on the pore sizes of mesoporous materials (M41S), surfactants with shorter hydrophobic chains were applied in the synthesis of ordered supermicroporous materials. However, due to the weak self-assembly abilities of such surfactants, the resulted materials usually possessed less ordered structures.[12–14] To overcome the problems mentioned above, three kinds of strategies were suggested, namely applying post-synthesis method,[15, 16] using a mixing templating system[17–19] and adopting elaborately designed template (*ω*-hydroxy-bolaform surfactants,[20, 21] Gemini surfactants,[22, 23] semifluorinated surfactants,[24] rigid-core surfactants,[25] ionic liquids[26, 27]). Although a few works have achieved the synthesis of ordered supermicroporous materials, the obtained materials were limited to silica-based materials. As a promising semiconductor, TiO_2_ has been widely used in the areas of catalyst supports, photocatalysis, sensors, solar cells and lithium-ion batteries.[28–32] Most of these applications require ordered pore structures and large surface areas.[33] Therefore, ordered mesoporous TiO_2_ is of particular interest in these fields. Great efforts have been made to produce ordered mesoporous TiO_2_ by using various template agents.[34–36] However, the successful synthesis of ordered supermicroporous titanias has rarely been reported yet. Chandra et al.[37] firstly introduced the synthesis of supermicroporous TiO_2_. Because titanium can easily form chelating complex with chelating ligands, two kinds of chelating agents, dodecyl-2-pyridinyl-methylamine and hexadecyl-2-pyridinyl-methylamine, were synthesized as the template agents. The synthesized materials showed enormous surface areas of 604–634 m^2^/g and narrow supermicropores of 1.4–1.68 nm. Unfortunately, the resulted TiO_2_ materials exhibited wormhole-like structures. It is of great significance to synthesize supermicroporous TiO_2_ with long-range order.

Our group has recently demonstrated the synthesis of ordered hexagonal supermicroporous silicas by using a kind of rosin-derived quaternary ammonium salt, dehydroabietyltrimethyl ammonium bromine (DTAB), as the template.[38] Such surfactant has a three-ring-phenanthrene-like hydrophobic group. Its strong self-assembly ability guarantees the construction of an ordered structure, while its smaller molecular size ensures the pore sizes of materials within the supermicropore range. In addition, rosin is a mixture of natural terpenoids extracted from pine trees. The renewable and low toxic properties of rosin-derived surfactant DTAB make it an environment-friendly reagent for achieving sustainable processes.

In the present study, templated by DTAB, ordered lamellar supermicroporous TiO_2_ was synthesized. To the best of our knowledge, there have been no reports on the preparation of ordered supermicroporous TiO_2_.

## Materials and methods

### 2.1 Materials

**Fig 1:**
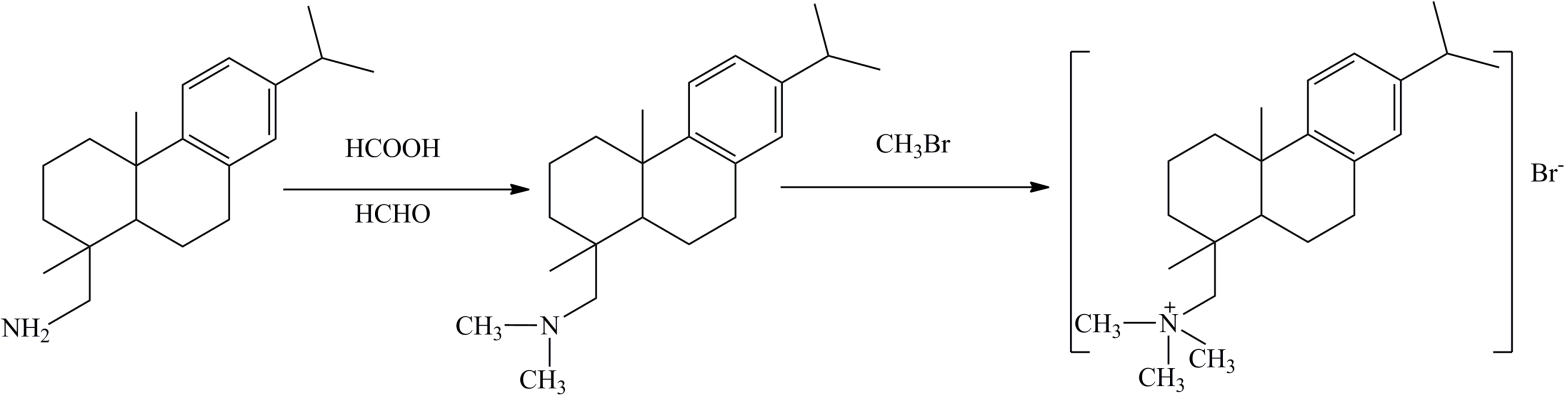
Synthesis route of dehydroabietyltrimethyl ammonium bromine.

Tetrabutyl titanate (TBOT, CP, ≥98%) was purchased from Shanghai Ling Feng Chemical Reagent Co., Ltd. Tetramethylammonium hydroxide (TMAOH, AR) was provided by Sinopharm Chemical Reagent Co., Ltd. The H_2_O_2_ (AR) was purchased from Shanghai Pilot Test Chemical Industry Co., Ltd. Dehydroabietyltrimethyl ammonium bromine (short for DTAB) was self-synthesized according to Fig 1. The detailed synthesis process of the surfactant has been described in literature[38]. The ^1^H NMR spectrum of DTAB is shown in S1 File.

### 2.2 Experiment

#### 2.2.1 Synthesis of ordered lamellar supermicroporous TiO_2_

The peroxotitanium acid precursor was synthesized according to the literature [39]. In a typical procedure, 4.356 g tetrabutyl titanate(12.5 mmol) was slowly added to the 25 g (1388.9 mmol) deionized water under stirring at 288 K. After stirring for 0.5 h, 15 g H_2_O_2_ (30% water solution, 132.3 mmol) was added dropwise. The resulting mixture was further stirred for 5 h until peroxotitanium acid ([TiO_2_(OH)(H_2_O)]OH) was obtained. 0.2 g (0.5 mmol), 0.3 g (0.75 mmol), 0.4 g (1.0 mmol), 0.5 g (1.25 mmol) of DTAB were dissolved in the solution of 20 g TMAOH (25% water solution, 27.6 mmol) and 20 g (1111.1 mmol) deionized water at 311 K, respectively. The obtained peroxotitanium acid was then added slowly into the above solution under stirring. The mole ratios of DTAB:TiO_2_ in the synthesis systems were 0.04:1, 0.06:1, 0.08:1 and 0.10:1. After stirring for 2 h, the suspension products were transferred into a Teflon-lined stainless steel autoclave and heated at 343 K, 353 K, 373 K or 393 K for 24 h. The solid was collected by suction filtration using Buchner funnel and washed with water and ethanol for several times to remove the impurities. The surfactants were removed by calciantion at 623 K for 2 h. The results are labeled as T-X-Y, where T denotes titania, X denotes the mole ratio of DTAB:TiO_2_, and Y denotes the hydrothermal temperature.

#### 2.2.2 Characterization

Powder XRD patterns were acquired on an X-ray diffractometer (D8 Focus, Bucker AXS Inc., Germany) with CuK*α* radiation (k = 0.15418 nm). The operating target voltage was 40 kV and the current was 40 mA. The suitable sample after grinding was scanned at 2*θ* ranging from 0.5° to 10° for small-angle XRD characterization and 10° to 80° for large-angle XRD characterization. Transmission electron microscopy (TEM) images of the pore structures were observed on a JEOL JEM-2100 instrument (JEOL Ltd., Japan) operated at an accelerating voltage of 200 kV. The samples were dispersed under ultrasonic in ethanol and then dropped with piette onto the carbon-coated copper grids prior to the measurement. Fourier transform infrared spectroscopy spectra of the samples were recorded with Nicolet iS10 spectrometer (Thermo Fisher Scientific Inc., USA). The samples were grinded with KBr and pressed to prepare the pellets. Then the spectra were conducted ranging from 4000 to 400 cm^-1^.

## Results and discussion

### 3.1 Effects of mole ratios of DTAB׃TiO_2_ on the microstructure characteristics of TiO_2_

**Fig 2:**
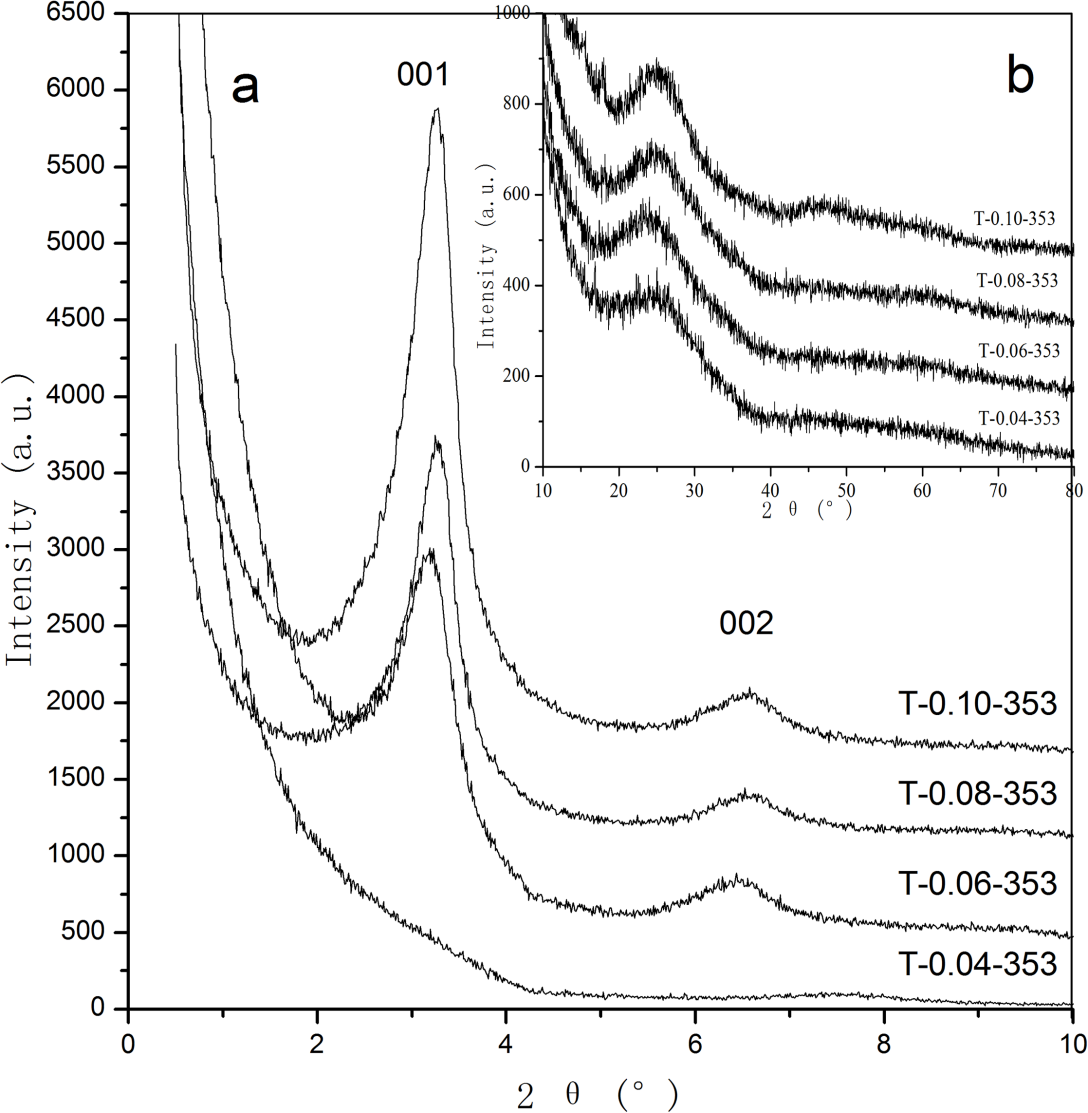
Small-angle (a) and large-angle (b) XRD patterns of as-synthesized supermicroporous titanias prepared with different DTAB dosages.

Fig 2A shows the small-angle XRD patterns of the as-synthesized samples prepared with various DTAB dosages. No diffraction peaks can be observed in the curve of the T-0.04–353 sample at 2*θ* ranging from 0.5° to 10°, which indicates that it is difficult to achieve ordered porous structure with an exiguity dosage of the template agent. As the mole ratio of DTAB:TiO_2_ increases to 0.06:1, the T-0.06–353 sample exhibits two diffraction peaks at 2*θ* = 3.22° (d_001_ = 2.74 nm) and 2*θ* = 6.49° (d_002_ = 1.36 nm), which can be related to the 001 and 002 reflections of lamellar phase. The interplanar spacing value of 001 plane is 2.74 nm, which is much smaller than that of the MCM-50,[40] suggesting that ordered lamellar supermicroporous structure is obtained. With the further adding of DTAB, diffraction peaks of the crystal planes are enhanced, revealing that the increase of DTAB concentration is beneficial to the improvement of the regularity of the products. As the mole ratio of DTAB:TiO_2_ is 0.10:1, the sample shows the highest and sharpest peaks, indicating that the highest ordering degree of the pore structures is obtained. When the mole ratio of DTAB:TiO_2_ exceeds 0.10:1, the surfactant cannot be completely dissolved in the reaction system. Therefore, the experiment was terminated. As can be seen from Fig 2B, all the samples shows a broad peak around 2*θ* = 25.00° together with a hump at 2*θ* ranging from 40.00° to 55.00°, suggesting that amorphous phase obtained after the hydrothermal treatment at 353 K for all the samples. There is no obvious difference among the large-angle XRD patterns of the samples, indicating that the amount of the surfactant has no significant effects on the crystalline structure of the samples.

### 3.2 Effects of hydrothermal temperatures on microstructure characteristics of TiO_2_

**Fig 3:**
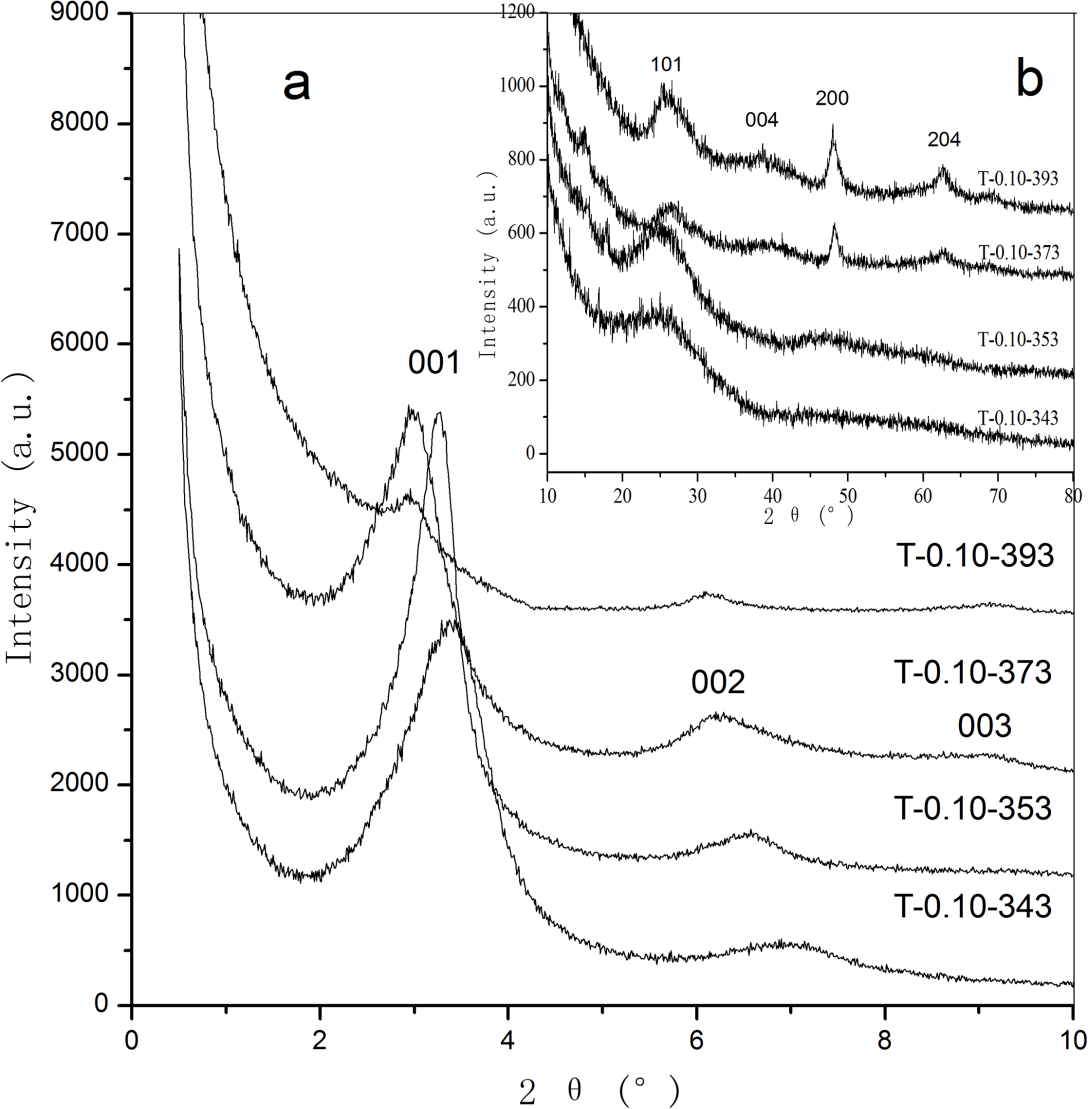
Small-angle (a) and large-angle (b) XRD patterns of as-synthesized supermicroporous titanias prepared at different hydrothermal temperatures.

Small-angle XRD patterns of as-synthesized titanias fabricated at different hydrothermal temperatures are shown in Fig 3A. When the hydrothermal temperatures are 343 and 353 K, both of the two samples of T-0.10–343 and T-0.10–353 reveal only two diffraction peaks of 001 and 002 crystal planes. As the hydrothermal temperature increases to 373 K, the sample T-0.10–373 exhibits three diffraction peaks of 001, 002 and 003 crystal planes, at 2θ = 2.98° (d_001_ = 2.96 nm), 6.23° (d_002_ = 1.42 nm) and 9.09° (d_003_ = 0.97 nm), suggesting that highly ordered lamellar supermicroporous structure is synthesized. When the hydrothermal temperature increases to 393 K, the sample T-0.10–393 still presents three diffraction peaks. However, the intensity of the peaks significantly decreases, indicating that the exorbitant hydrothermal temperature will weaken the ordering degree of the products. It is worth noting that the small-angle diffraction peaks of the samples shift to the left as a function of increasing hydrothermal temperature, suggesting the expansion of unit-cell upon hydrothermal treatment at higher temperature. Fig 3B shows the large-angle XRD patterns of as-synthesized samples treated at different hydrothermal temperatures. As the hydrothermal temperature increases, the crystallinity of the as-synthesized titanias increases accordingly. When the hydrothermal temperature is 373 K, the sample T-0.10–393 shows four board peaks at 2θ = 26.36° (101), 38.88° (004), 48.25° (200) and 62.93° (204), which can be related to the semicrystallized anatase phase (JCPDS, No. 21–1272). Upon the further increase of hydrothermal temperature to 393 K, the intensity of the diffraction peaks for sample T-0.10–393 is enhanced slightly. Unfortunately, the regularity of the pore structure decays at such higher hydrothermal temperature.

### 3.3 Effects of calcinations on microstructure characteristics of TiO_2_

**Fig 4:**
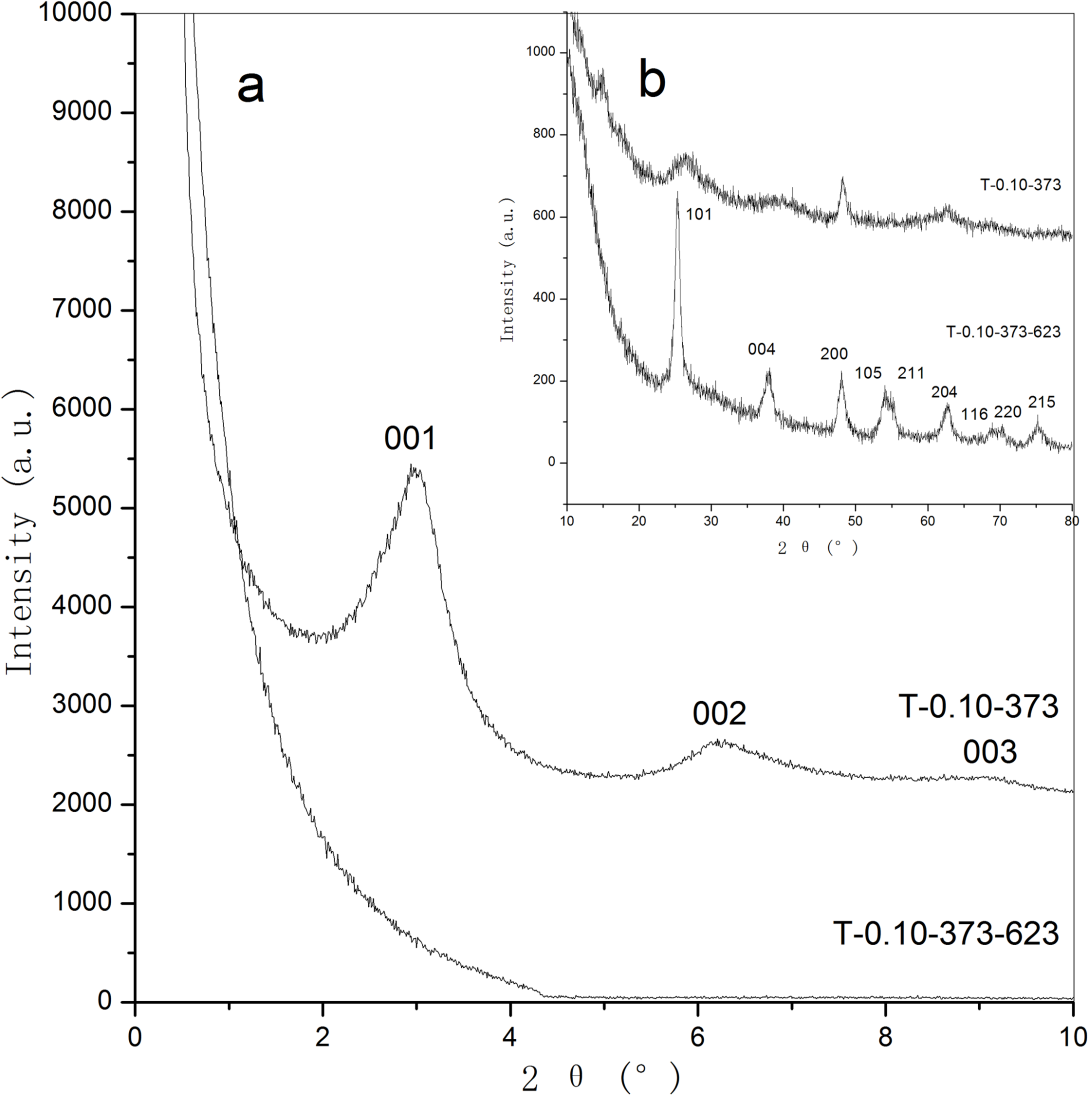
Small-angle (a) and large-angle (b) XRD patterns of as-synthesized and calcined supermicroporous titanias.

Fig 4A illustrates the small-angle XRD patterns of the as-synthesized and calcined supermicroporous titanias. Just like all the other lamellar phase materials,[40, 41] the samples we synthesized also possess poor heat stability. After the thermal treatment at 623 K, no diffraction peaks can be observed in the small-angle XRD pattern due to the complete collapse of the pore channels. As can be seen in Fig 4B, the as-synthesized sample T-0.10–373 shows broad anatase peaks, suggesting that semicrystallized anatase phase is obtained. After the calcination at 623 K, the sample T-0.10-373-623 displays much sharper and stronger peaks which can be indexed to an anatase phase with high crystallinity.

### 3.4 The FTIR characterizations on surfactant and TiO_2_

**Fig 5:**
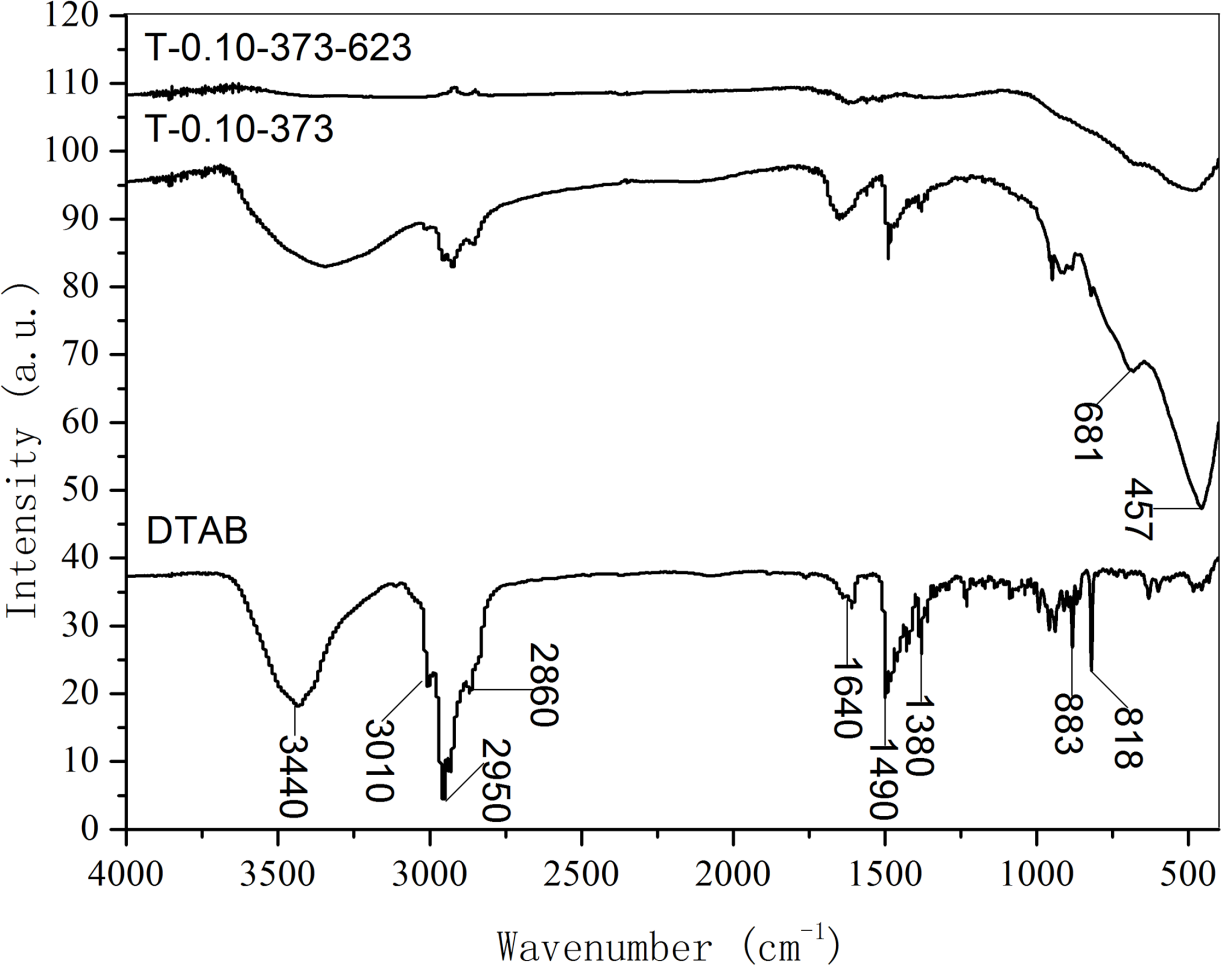
FTIR spectra of surfactant, as-synthesized and calcined supermicroporous titanias.

Fig 5 plots the FTIR spectra of surfactant, as-synthesized and calcined samples. As for the DTAB surfactant, the peak at 3010 cm^-1^ belongs to the stretching vibration of Ar C-H, and the peak at 1490 cm^-1^ belongs to the stretching vibration of Ar C = C. The peaks at 883 and 818 cm^-1^ are due to the asymmetric tri-substituted benzene ring. The bands at 2950, 2860 and 1380 cm^-1^ can be assigned to the stretching vibration of -CH_3_- or -CH_2_-. The bands at 3440 and 1640 cm^-1^ are caused by the vibration of adsorbed water. It can be seen from the as-synthesized sample T-0.10–373, the bands at 681 and 457 cm^-1^ can be related to the Ti-O stretching vibration of anatase titania. The peaks with reduced intensity belonging to DTAB can be observed in the as-synthesized sample, suggesting that the DTAB template agent is incorporated in the pore of TiO_2_. After the calcination at 623 K, the bands caused by anatase titania still can be observed. Meanwhile, the peaks belonging to DTAB can barely be observed, indicating that the DTAB surfactant is removed by the calcination.

### 3.5 The TEM characterizations on TiO_2_

**Fig 6:**
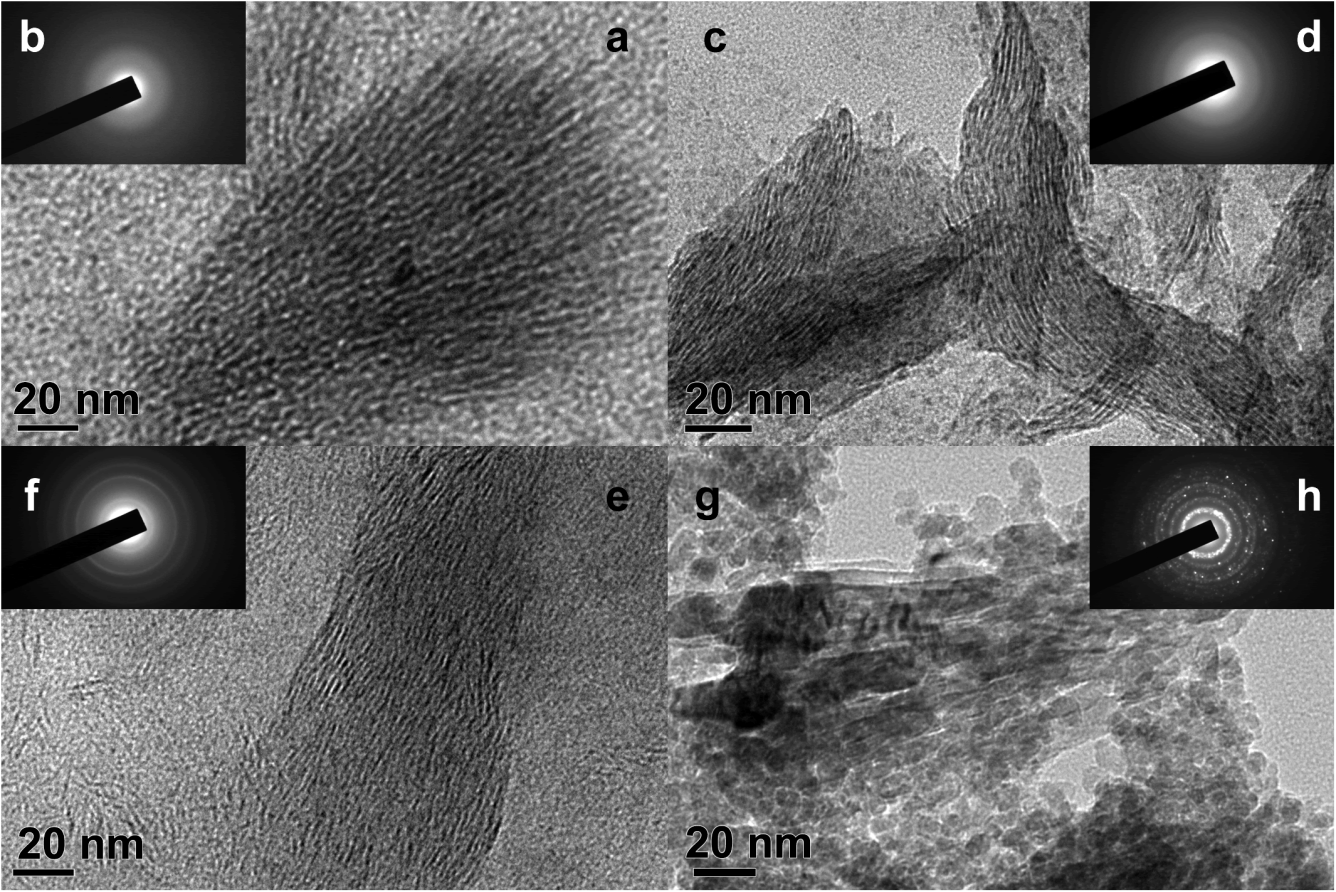
TEM images (a, c, e, g) and electron diffraction patterns (b, d, f, h) of as-synthesized and calcined supermicroporous titanias (a, b: T-0.06–353, c, d: T-0.10–373, e, f: T-0.10–393, g, h: T-0.10-373-623).

Fig 6 presents the TEM characterizations of the as-synthesized and calcined titanias. The sample T-0.06–353 (Fig 6A) shows radial pore structure with wormhole-like channels in some regions, which can be related to a lamellar phase without long-range order. After optimizing the reaction parameters of DTAB dosage and hydrothermal temperature, the sample T-0.10–373 (Fig 6C) displays parallel channels with long-range order, suggesting a practically ordered lamellar phase is obtained. It can be seen that the pore size and the pore wall thickness are about 2.0 nm and 1.0 nm, respectively. As for the sample T-0.10–393 (Fig 6E), the collapse phenomenon of pore channels can be observed in partial regions as the hydrothermal temperature increases to 393 K. After the calcination at 623 K, the ordered pore channels collapse completely. It can be seen that the tunnel of the sample T-0.10-373-623 (Fig 6G) is formed by the simple accumulation of nanometer grains. Such results are coincident with the small-angle XRD characterizations. Fig 6B shows the electron diffraction pattern obtained from a selected area of Fig 6A. No diffraction rings can be observed in the pattern, indicating the amorphous phase is formed in this sample. Fig 6D shows ambiguous diffraction rings, suggesting the semicrystallized anatase phase is obtained after the hydrothermal treatment at 373 K. With the increase of hydrothermal temperature from 373 K to 393 K, the diffraction rings (Fig 6F) become clearer, suggesting the increase of the crystallinity. Upon the calcination at 623 K, the diffraction rings become more distinct, and some bright spots can also be observed in Fig 6H, which may be derived from the presence of highly crystallized nanometer grains. Such result is also in good agreement with the results of large-angle XRD characterizations.

## Conclusions

In summary, ordered lamellar supermicroporous titania has been synthesized by using rosin-derived quaternary ammonium salt as template via a hydrothermal process. The results indicate that the template agent: titanium source molar ratio in the synthesis system and the hydrothermal temperature have great influences on the microstructure characteristics of the titanias. The increase of template agent results in the improvement of regularity of pore structures, but has no significant effects on the crystalline structures. The increase of the hydrothermal temperature is beneficial to enhancing the crystallinity of the samples. However, the exorbitant hydrothermal temperature will undermine the regularity of pore structures. As the mole ratio of DTAB:TiO_2_ is 0.10:1 and the hydrothermal temperature is 373 K, the as-synthesized sample reveals the pore structure with the highest level of regularity, as well as pore wall with semicrystallized anatase phase. The pore size of the sample is about 2.0 nm, and the pore wall thickness is about 1.0 nm.

## Supporting information

S1 File The ^1^H NMR spectrum of DTAB. (PDF)
