# Supplementary figures and images for: Ordered lamellar supermicroporous titania templating by rosin-derived quaternary ammonium salt
Source: PLoS One. 2017 Jun 30;12(6):e0180178. doi: 10.1371/journal.pone.0180178 (PMC5493357; doi:10.1371/journal.pone.0180178)

2015-2

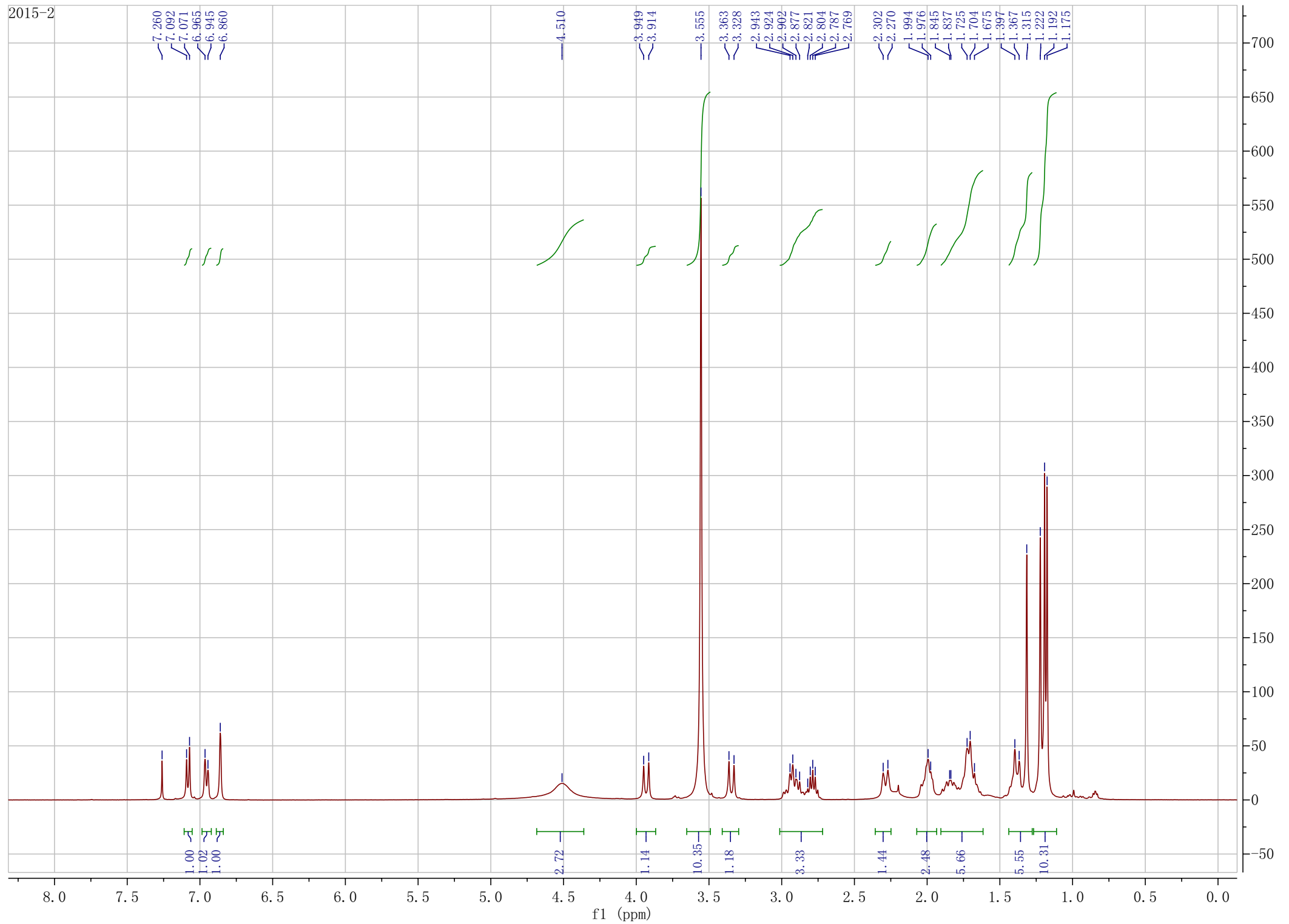

Supplement: S1 File — (PDF) [file pone.0180178.s001.pdf]
